# Maternal and Neonatal Outcomes of Sub-clinical Hypothyroidism Treated With Levothyroxine in Pregnancy: A Retrospective Cohort Study

**DOI:** 10.7759/cureus.45352

**Published:** 2023-09-16

**Authors:** Nastaran Safavi Ardabili, Farinaz Rahimi, Amene Ranjbar, Farideh Montazeri, Fatemeh Darsareh

**Affiliations:** 1 Department of Midwifery, Ardabil Branch, Islamic Azad University, Ardabil, IRN; 2 Mother and Child Welfare Research Center, Hormozgan University of Medical Sciences, Bandar Abbas, IRN; 3 Fertility and Infertility Research Center, Hormozgan University of Medical Sciences, Bandar abbas, IRN; 4 Mother and Child Welfare Research Center, Hormozgan University of Medical Sciences, Bandar abbas, IRN

**Keywords:** neonatal outcomes, maternal outcomes, childbirth, pregnancy, sub-clinical hypothyroidism

## Abstract

Introduction: The effect of sub-clinical hypothyroidism (SCH) in pregnancy has been controversial. Furthermore, the impact of levothyroxine replacement on improving outcomes in pregnant women with SCH is unknown. This study aimed to assess the maternal and neonatal outcomes of pregnant women with SCH who were treated with levothyroxine replacement.

Methods: This retrospective chart review was conducted at a tertiary hospital in Iran between 2020 and 2022. All pregnant women who had given birth during the study period were recruited. Those who did not have thyroid function test results within 10-12 weeks, as well as those with SCH who did not have levothyroxine replacement, were excluded. The subjects were divided into two groups based on the 2017 American Thyroid Association (ATA) criteria: non-SCH (TSH values 0.27-2.5 mIU/L) and SCH (TSH values more than 4.0 mIU/L). The demographic, obstetric, maternal, and neonatal outcomes of both groups were compared. The Chi-square test was used to compare the categorical variables. Binary logistic regression was used to assess differences in categorical variables.

Results: With a frequency of 10.5%, 935 women out of 8888 were diagnosed with SCH. In terms of age, educational level, living residency, medical insurance, access to prenatal care, and smoking status, there were no differences between the two groups. In terms of gestational age, parity, onset of labor, history of infertility, hypertension, cardiovascular disease, anemia, and overt diabetes, there were no differences between the two groups; however, gestational diabetes was more common in those with SCH. Compared with the non-SCH group, the prevalence and risks of gestational diabetes [19.8 vs. 14.2, odds ratio (OR) = 1.14, 95% confidence interval (CI) = 1.72-3.95] were significantly higher in the SCH group after controlling for confounding factors. There were no differences in neonatal outcomes between the two groups.

Conclusions: Except for gestational diabetes, we found no significant adverse events in terms of maternal and neonatal outcomes among women with SCH who were treated with levothyroxine.

## Introduction

The negative impact of overt hypothyroidism on the prenatal outcomes of pregnant women has been demonstrated in several studies [1,2]. However, the effect of sub-clinical hypothyroidism (SCH) in pregnancy has been controversial. SCH has been characterized as an elevated thyroid-stimulating hormone (TSH) with normal free thyroxin [3]. In several studies, SCH has been linked to an increased risk of negative maternal and neonatal outcomes in pregnancy, such as pregnancy loss, preterm delivery, gestational diabetes, pregnancy-induced hypertension, preeclampsia, sudden placental abruption, premature rupture of membranes, intrauterine growth retardation, low birth weight, and neonatal death [4,5]. Furthermore, high TSH levels in pregnant women have been linked to an increased risk of neurocognitive deficits in their children [6]. Other studies, however, have found no negative effects linked to SCH [7]. Furthermore, the impact of levothyroxine replacement on improving outcomes in pregnant women with SCH is unknown [8]. The goal of this study was to compare the maternal and neonatal outcomes of pregnant women with SCH who were treated with levothyroxine replacement to those of women without SCH.

## Materials and methods

This retrospective chart review was conducted at a tertiary hospital in Iran between 2020 and 2022. Informed consent is waived because it is not practicable to obtain consent from large numbers of patients for a retrospective chart review study; generally, it is also not appropriate to attempt to contact those patients to tell them about the study retrospectively. Statistical analysis was performed with patient anonymity. Data were extracted from the "Iranian Maternal and Neonatal Network (IMaN Net)," a valid national system, by trained collectors using electronic patient records.

All pregnant women who had given birth during the study period were recruited as potential participants. Those who did not have thyroid function test results within 10-12 weeks, as well as those with SCH who did not have levothyroxine replacement, were excluded, as were those who had incomplete data. The subjects were divided into two groups based on the 2017 American Thyroid Association (ATA) [9] criteria: non-SCH (TSH values 0.27-2.5 mIU/L) and SCH (TSH values more than 4.0 mIU/L). Demographic factors (age, education, living residency, access to prenatal care, insurance, and smoking), obstetrical factors (gestational age, parity, history of infertility), maternal disease (anemia, chronic hypertension, cardiovascular disease, diabetes) maternal outcomes (preeclampsia, gestational diabetes, abruption, fetal distress, meconium, mode of delivery, onset of labor, shoulder dystocia, intrauterine fetal retardation, and intrauterine fetal death) and neonatal outcomes (newborn asphexia, neonatal intensive care unit, neonatal death, and neonatal congenital malformation) of the two groups were compared.

SPSS 19.0 (IBM Corp., Armonk, NY) was used to analyze the data. Data were presented as range or frequency. The chi-square test was used to compare the categorical variables. Binary logistic regression was used to assess differences in categorical variables. P = 0.05 (two-sided) was considered statistically significant.

## Results

Of the 9045 women who had childbirth during the study period, 157 pregnant women were excluded from our study because they did not have a TSH test in the first trimester of pregnancy or had not received levothyroxine replacement during pregnancy. With a frequency of 10.5%, 935 women out of 8888 were diagnosed with SCH. In terms of age, educational level, living residency, medical insurance, access to prenatal care, and smoking status, there were no differences between the two groups (Table 1).

**Table 1:** Comparison of maternal characteristics of women with SCH and non-SCH Data are presented as n (%). SCH: sub-clinical hypothyroidism.

Demographic characteristics	Non-SCH (n=7952)	SCH (n=936)	P-value
Age (years)			0.061
13–19	158 (2)	15 (1.6)	
20–34	6528 (82.1)	741 (79.2)	
35 and above	1266 (15.9)	180 (19.3)	
Educational level			0.051
Illiterate	517 (6.5)	40 (4.3)	
Elementary	2468 (31.2)	252 (26.9)	
High school/diploma	3651 (45.9)	438 (46.8)	
Advanced	1304 (16.4)	206 (22)	
Residency place			0.053
Urban	5243 (65.9)	655 (70)	
Rural	2709 (34.1)	281 (30)	
Access to prenatal care			0.097
Yes	7714 (97)	919 (98.2)	
No	238 (3)	17 (1.8)	
Medical insurance			0.171
Yes	7004 (88.1)	811 (86.7)	
No	948 (11.9)	125 (13.3)	
Smoking			0.746
Yes	61 (0.8)	11 (1.1)	
No	7891 (99.2)	925 (98.9)	

In terms of gestational age, parity, onset of labor, history of infertility, hypertension, cardiovascular disease, anemia, and overt diabetes, there were no differences between the two groups; however, gestational diabetes was more common in those with SCH (Table 2).

**Table 2:** Comparison of medical and obstetrical characteristics of women with SCH and non-SCH Data are presented as n(%). SCH: Sub-clinical hypothyroidism.

Variables	Non-SCH (n=7952)	SCH (n=936)	P-value
Gestational age			0.134
Less than 37 weeks	1105 (13.9)	140 (15)	
37–40 weeks	5745 (72.2)	691 (73.8)	
40^+1^–41 weeks	928 (11.7)	86 (9.2)	
More than 41 weeks	174 (2.2)	19 (2)	
Parity			0.970
Primiparous	2241 (28.2)	266 (28.4)	
Multiparous (2-5)	5498 (69.1)	644 (68.8)	
Grand multiparous (6 parity or more)	213 (2.7)	26 (2.8)	
Onset of labor			0.118
Spontaneous	4570 (57.5)	528 (56.4)	
Labor induction	1864 (23.4)	205 (21.9)	
Cesarean before the onset of labor	1518 (19.1)	203 (21.7)	
Infertility			0.999
No	7927 (99.7)	933 (99.7)	
Yes	25 (0.3)	3 (0.3)	
Chronic hypertension			0.717
No	7873 (99)	917 (98)	
Yes	79 (1)	19 (2)	
Anemia			0.607
No	7725 (97.1)	906 (96.8)	
Yes	227 (12.9)	30 (13.2)	
Cardiovascular disease			0.879
No	7873 (99)	920 (98.3)	
Yes	79 (1)	16 (1.7)	
Diabetes			< 0.001
No	6793 (85.4)	747 (79.8)	
Gestational diabetes	1132 (14.2)	186 (19.8)	
Overt diabetes	27 (0.3)	3 (0.3)	

There were no differences in maternal and neonatal outcomes between the two groups, as shown in Table 3.

**Table 3:** Comparison of maternal and neonatal outcomes of women with SCH and non-SCH Data are presented as n (%). SCH: sub-clinical hypothyroidism.

Variables	Non-SCH (n=7952)	SCH (n=936)	P-value
Preeclampsia			0.834
No	7437 (93.5)	874 (93.4)	
Yes	515 (6.5)	62 (6.6)	
Placenta abruption			0.769
No	7695 (96.8)	908 (97)	
Yes	257 (3.2)	28 (3)	
Meconium fluid			0.673
No	6985 (87.8)	818 (87.4)	
Yes	967 (12.2)	118 (12.6)	
Method of delivery			0.967
Normal vaginal delivery	5236 (65.8)	612 (65.4)	
Instrumental delivery	73 (0.9)	10 (1.1)	
Cesarean section	2643 (33.2)	314 (33.5)	
Postpartum hemorrhage			0.199
No	7811 (98.2)	914 (97.6)	
Yes	141 (1.8)	22 (2.4)	
Low birth weight (Less than 2500 g)			0.865
No	6864 (86.3)	806 (86.1)	
Yes	1088 (13.7)	130 (13.9)	
Macrosomia (More than 4000 g)			0.601
No	7786 (97.9)	919 (98.2)	
Yes	166 (2.1)	17 (1.8)	
Intrauterine growth retardation			
No	7701 (96.8)	898 (95.9)	0.144
Yes	251 (3.2)	38 (4.1)	
Intrauterine fetal death			0.703
No	7882 (99.1)	930 (99.4)	
Yes	70 (0.9)	6 (0.6)	
Childbirth injury			0.921
No	7938 (99.8)	935 (99.9)	
Yes	14 (0.2)	1 (0.1)	
Shoulder dystocia			0.989
No	7902 (99.4)	930 (99.4)	
Yes	50 (0.6)	6 (0.6)	
Neonatal congenital malformation			0.191
No	7865 (98.9)	921 (98.4)	
Yes	87 (1.1)	15 (1.6)	
Need for neonatal resuscitation			0.157
No	7400 (93)	856 (91.6)	
The primary levels of resuscitation	380 (4.7)	51 (5.4)	
Advanced levels of resuscitation	172 (2.3)	29 (3)	
Newborn asphyxia			0.106
No	7889 (99.2)	926 (99)	
Yes	63 (0.8)	10 (1)	
Neonatal intensive care unit admission			0.196
No	7410 (93.2)	877 (93.7)	
Yes	542 (6.8)	59 (6.3)	
Newborn death			0.994
No	7928 (99.7)	933 (99.7)	
Yes	24 (0.3)	3 (0.3)	

Since gestational diabetes was the only measure outcome that differed between the SCH and non-SCH groups, we evaluated the association of SCH with gestational diabetes as shown in Table 4. As shown in Table 4, based on the 2017 ATA guidelines, compared with the non-SCH group, the prevalence and risks of gestational diabetes [19.8 vs. 14.2, odds ratio (OR) = 1.14, 95% confidence interval (CI) = 1.72-3.95] were significantly higher in the SCH group after controlling for confounding factors.

**Table 4:** Association of maternal SCH with gestational diabetes OR: odds ratio, SCH: sub-clinical hypothyroidism.

Outcome	Non-SCH (%)	SCH (%)	OR (95% CI)	P-value	Adjusted OR (95% CI)	P-value
Gestational diabetes	14.2%	19.8%	2.69 (1.75–5.81)	<0.001	2.14 (1.72–3.95)	<0.001

## Discussion

SCH is more common than overt hypothyroidism during pregnancy, with rates ranging from 10% to 28% in iodine-sufficient areas [10]. This wide range can be explained by differences in study design, diagnostic criteria, population characteristics, and geographic factors. In our study population, SCH occurred at a rate of 10.5%.

Some studies found that SCH was linked to several adverse events of pregnancy and childbirth, such as pregnancy-induced hypertension, preeclampsia, preterm childbirth, and sudden placental abruption; however, these associations have not been replicated in more recent studies [11]. These inconsistencies are most likely due to differences in the diagnostic criteria for SCH (different TSH cutoffs) used in different studies. As a result, how to define SCH in pregnancy has become increasingly contentious in recent years [12]. Using different diagnostic guidelines for SCH resulted in different conclusions. A study that used both the 2011 ATA guidelines and the 2017 ATA guidelines to evaluate the negative impact of SCH on pregnancy outcomes found that maternal SCH identified by the 2017 ATA guidelines was associated with higher rates of total adverse maternal and neonatal outcomes than maternal SCH diagnosed by the 2011 ATA guidelines. According to them, the higher level of TSH was more attributed to maternal outcome [13].

In contrast to several studies [4,5], we found no significant adverse events in terms of maternal and neonatal outcomes among women with SCH who were treated with levothyroxine. Gestational diabetes was the only condition that occurred more frequently in those with SCH. The most likely explanation for this disparity is that we chose those who had received levothyroxine as a study population. The main question in the care of pregnant women with SCH is whether to treat or not to treat. As a result, the majority of recent research has concentrated on determining the magnitude of the effect of thyroid hormone therapy on improving maternal and neonatal outcomes, although some studies showed no beneficial effect of levothyroxine replacement on prenatal outcomes. For example, a randomized clinical trial by Nazarpour et al. discovered that LT4 therapy had no overall beneficial effect [14]. Another study by Casey et al. discovered no difference in maternal and neonatal outcomes following levothyroxine treatment of SCH during pregnancy [15]. A recent meta-analysis found that taking levothyroxine during pregnancy reduced the risk of miscarriage and neonatal death in women with SCH. There was no link found between levothyroxine treatment and outcomes during labor and delivery [16]. The role of levothyroxine in improving maternal and neonatal outcomes and long-term outcomes is still being investigated. More research is needed in this area. The limitation of the study is that we did not evaluate the long-term outcomes.

## Conclusions

Except for gestational diabetes, we found no significant adverse events in terms of maternal and neonatal outcomes among women with SCH who were treated with levothyroxine. The current study's findings support levothyroxin's beneficial role in reducing maternal and neonatal complications. More research in this field is necessary to better understand the pregnancy and childbirth outcomes of SCH and the impact of levothyroxine replacement on avoiding adverse events. While waiting for the results of ongoing efficacy trials and the conduct of larger trials of levothyroxine therapy in high-risk women with SCH during pregnancy, clinicians and patients must engage in open and shared decision-making.
